# The role of elastography in the assessment of thyroid nodules in acromegaly

**DOI:** 10.55730/1300-0144.5585

**Published:** 2022-12-21

**Authors:** Hakan DÜĞER, Muhammed KIZILGÜL, Pınar AKHANLI, Murat ÇALAPKULU, Hayri BOSTAN, Sema HEPŞEN, Ümran GÜL, Muhammed Erkam SENCAR, Erman ÇAKAL, Bekir UÇAN

**Affiliations:** Health Sciences University, Dışkapı Yıldırım Beyazit Training and Research Hospital, Department of Endocrinology and Metabolism, Ankara, Turkey

**Keywords:** Elastography, thyroid nodules, acromegaly

## Abstract

**Background/aim:**

Nodular thyroid disease is a frequent finding seen in patients with acromegaly. Ultrasound-elastography (US-E) appears to be a helpful tool for the diagnosis of thyroid cancer. The aims of this study were to evaluate thyroid nodules in acromegaly and to assess the diagnostic accuracy of US-E in detecting thyroid cancer in this population.

**Material and methods:**

US-E was applied to 166 nodules detected in 102 acromegalic patients and to 105 nodules found in 95 nonacromegalic subjects. The lesions were classified according to the elasticity scores (ES) as soft (ES 1–2) or hard (ES 3–4).

**Results:**

Mean age was 55.1 ± 12.47 years [59 (58%) women]. The prevalence of hard nodules (ES 3 and 4) was significantly higher in the group of acromegalic patients than in control subjects (48% to 20%, p < 0.001). Mean ES was higher in patients with acromegaly (2.45 to 2.22, p: 0.001), however, the mean strain index (SI) was similar between groups (1.53 to 1.65, p: 0.204).

**Conclusion:**

Thyroid nodules in acromegaly patients have a higher elasto score and the prevalence of hard nodules is higher in active disease. However, increased stiffness of nodules by US-E in patients with acromegaly does not seem to estimate the malignancy of the nodules.

## 1. Introduction

Acromegaly is a chronic systemic disease caused by excess levels of growth hormone (GH) and consequently insulin-like growth factor-1 (IGF-1) that is caused by a GH-secreting pituitary adenoma in the majority of the cases [1]. It is known that IGF-1 is a growth factor for thyroid tissue in vitro [2]. IGF-1 levels are correlated with thyroid volume in patients with acromegaly [3]. The synthesis of protein and deoxyribonucleic acid (DNA) in thyrocytes as well as the proliferation and differentiation of these cells are stimulated by IGF-1 [4,5]. Sustained exposure to increased levels of IGF-1 may have a role in the occurrence of thyroid nodules and elevated IGF-1 levels are related to the presence of thyroid nodules in patients with acromegaly [6]. Nodular thyroid disease is a common finding in patients with acromegaly. According to a recent study; 69% of acromegaly patients have nodular thyroid disease [7]. Elastography is a new technique that provides the assessment of tissue elasticity by ultrasound guidance. Malignant lesions often have greater stiffness compared to normal ones [8]. Although the functional and ultrasonographic characteristics of thyroid nodules in acromegaly patients have been well-documented, there are scarce data related to the elastographic features of these lesions. A recent study reported a higher prevalence of hard thyroid nodules in patients with acromegaly, but this was not associated with an increased rate of malignancy [9].

This study aimed to evaluate the elastic properties of acromegaly-associated thyroid nodules with elastography and to investigate the diagnostic accuracy of this technique in detecting thyroid malignancy using cytological analysis as a reference.

## 2. Materials and methods

We analyzed all data retrospectively for 206 consecutive patients with acromegaly followed up after surgery in the Endocrinology and Metabolism Department of Dışkapı Training and Research Hospital. We excluded 35 patients who do not have ultrasonographic evaluation. Twelve patients passed away during follow-up and were therefore excluded from the study. The study protocol was reviewed and approved by the Health Sciences University, Dışkapı Training and Research Hospital Institutional Review Board. All investigations were performed in accordance with relevant guidelines and regulations (2015 American Thyroid Association guidelines). A total of 159 acromegaly patients had an ultrasonographic evaluation and 102 of them had nodular thyroid diseases. One hundred and five out of 166 nodules, which were performed ultrasound-elastography (US-E) in the acromegaly group had fine needle aspiration cytology (FNAC). However, 96 out of 105 nodules in the control group, which consisted of 95 patients, had FNAC.

Acromegaly was diagnosed according to the presence of an increased serum IGF-1 concentration with typical clinical manifestations, insufficient suppression of serum GH after a glucose load, and the presence of a pituitary tumor.

A normal age- and sex-adjusted normal ranges of circulating IGF-1 levels and a postoperative random GH level of <1 ng/mL or GH level of <0.4 ng/mL after a glucose load were used as the remission criteria. Patients were categorized into the control group when IGF-1 was within age- and sex-adjusted normal ranges and were otherwise categorized into the active disease group [10].

The GH and IGF-1 concentrations were measured by using chemiluminescence on an IMMULITE 2000 Xpi (Siemens Health-care Diagnostics Inc). Serum IGF-1 levels were compared with the age- and sex-adjusted normal reference values. All patients and controls were euthyroid.

### 2.1. Thyroid US and US elastography

Thyroid ultrasound and elastographic examination were performed before FNAC using an EUB-7000 HV scanner (Hitachi Medical Corporation, Tokyo, Japan) with a 6- to 13-MHz linear array transducer. The volume of each thyroid lobe was calculated by the ellipsoid model formula (length × thickness × width × 0.52) [11].

The nodules were evaluated in terms of echogenicity, size, regularity of margin, the presence and nature of the halo, the presence of calcifications, and the blood flow pattern. Itoh’s elasticity score scale was used for the measurement of elastography score (ES) according to different nodule color patterns [12]. The US elastogram was displayed over the B-mode image in a color scale depending on the magnitude of strain: red (soft tissue), green (inter-mediate degree of stiffness), and blue (hard, elastic tissue). The lesions were classified into four classes of hardness according to their colors. We used the longitudinal view for measurement and US-E was performed only for nodules larger than 10 mm.

An area manually along the borderline of the nodule and a similar-sized area beside the nodule in thyroid tissue were selected as a reference. The strain index (SI) was measured automatically by the software. The likelihood of malignancy increases with an increase in the strain ratio.

The average of three consecutive measurements was taken into account. Three experienced endocrinologists performed all the measurements.

### 2.2. Cytopathological diagnosis

Cytological examination of material obtained by FNAC was used as a reference standard to establish the benign/malignant nature of the lesion. All FNAC were classified by 2008 Bethesda categories: nondiagnostic, benign, atypia of undetermined significance/follicular lesion of undetermined significance, follicular neoplasm (FN), suspicious for malignancy, and malignant [13].

### 2.3. Statistical analysis

All statistical analyses were carried out using the JMP 14.0.1 software (SAS Institute). Mean ± SD and counts and proportions were used for the expression of quantitative data and categorical data, respectively. The normality of distribution was examined by using the Kolmogorov-Smirnov or Shapiro-Wilk W test. The X^2^ or Fisher exact test was used when the variables were categorical. The t-test and the Mann-Whitney U test was used for normally and nonnormally distributed continuous variables, respectively. A p value of <0.05 was accepted as statistically significant.

## 3. Results

One hundred and two acromegalic patients and 95 control subjects were enrolled in the study. The mean age was similar between groups (55.08 ± 12.47 to 51.77 ± 13.65, p = 0.078). The ratio of female participants was also similar between groups (57% to 67%, p = 0.063). In acromegaly patients,162 thyroid nodules were found and the number of lesions in a single patient varied from 1 to 13. The mean thyroid volume was higher in the acromegaly group (33.1 ± 31.2 mL to 21.42 ± 11.12, p < 0.001). The mean size of the nodules was 9.39 ± 6.42 mm (range 3–37). The characteristics of patients with acromegaly and US features of both groups are shown in (Table 1) and (Table 2), respectively.

In acromegalic patients, FNAC was performed in 105/166 (63%) of the nodules, namely 58/76 (76%) of the ES 3–4 nodules and 47/90 (52%) of the ES 1–2 nodules. FNAC was not carried out in 8 hard nodules due to US characteristics of benignity (isoechogenicity, regular margins, type I or II flow pattern, and absence of micro-calcifications), small size or patients’ refusal. In the control group, FNAC was performed in 96/105 (91%) of the nodules, namely 20/22 (91%) of the hard nodules and 76/83 (92%) of the soft nodules.

**Figure 1:**
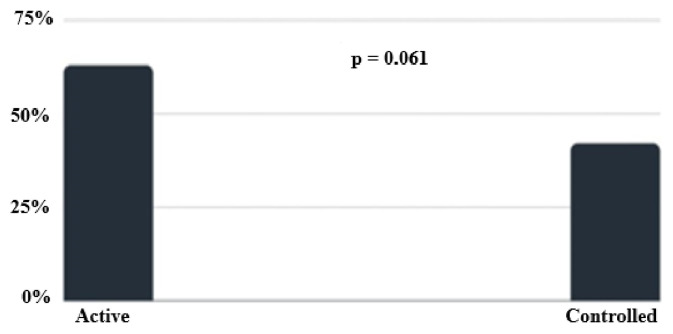
The prevalence of hard nodules according to disease activity in patients with acromegaly.

All nodules with ES of 3 or 4 that did not undergo FNAC were evaluated by US and US-E for at least 12 months. US-E evaluation was performed in 166 nodules in 102 acromegaly patients. The disease was active in 22% of these patients. In all, 90 soft and 76 hard nodules were found. In detail, the ES was 1 for 7 nodules (4%), 2 for 83 lesions (50%), 3 for 70 nodules (42%), and 4 for the remaining 6 (4%). Although the prevalence of hard nodules (ES 3–4) in patients with the active disease was higher than that of soft nodules (ES 1–2), it did not reach a statistically significant point (65% to 43%, p = 0.061) (Figure 1).

**Figure 2:**
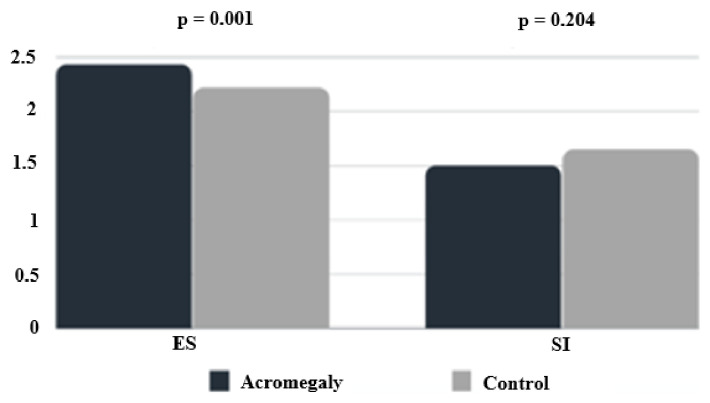
Comparison of elasto score (ES) and strain index (SI) between groups.

In nonacromegalic goitrous patients, US-E evaluation was performed in 105 nodules in 95 patients. In all, 83 soft nodules and 22 hard lesions were found. In detail, the ES was 1 for 0 nodules (0%), 2 for 83 lesions (79%), 3 for 20 nodules (19%), and 4 for the remaining 2 (2%). The prevalence of hard nodules (ES 3 and 4) was significantly higher in the group of acromegalic patients than in control subjects (48% to 20%, p < 0.001) (Table 3). Mean ES was higher in patients with acromegaly (2.45 to 2.22, p = 0.001). However, the mean strain index was similar between groups (1.53 to 1.65, p = 0.204). The comparison of elasto score and strain index between groups is shown in (Figure 2).

### 3.1. Cytopathological diagnosis

FNAC was performed in 105/166 (63.2%) and 96/105 (91.4%) of the nodules in the acromegaly and control groups, respectively. In the acromegaly group, 105 of 166 thyroid nodules (63.2%) underwent FNAC (results: 70 benign, 16 nondiagnostic, 13 atypia of unknown significance, 3 follicular neoplasm, 1 suspicious for malignancy, and 2 papillary thyroid cancer [PTC]), from which six nodules in 5 patients underwent thyroidectomy; PTC was confirmed by surgical pathology for all cases. On the other hand, in the control group, 96 of 105 thyroid nodules (91.4%) underwent FNAC (results: 77 benign, 9 nondiagnostic, 5 atypia of unknown significance, 1 follicular neoplasm, 2 suspicious for malignancy, and 2 PTC), in which six of them underwent thyroidectomy; PTC was confirmed by surgical pathology in 4 cases. Two of the 58 hard nodules detected in our acromegalic patients were found to be malignant at the cytological examination, while 2 out of 20 hard nodules aspirated in control subjects turned out to be malignant.

## 4. Discussion

Our study found that the prevalence of hard nodules and mean elasto score were significantly higher in the group of acromegalic patients than in control subjects. However, the prevalence of hard nodules was similar between active and controlled acromegaly patients. Additionally, the higher prevalence of hard nodules did not increase the malignancy rate in these patients. In one of the first studies on this subject, Andrioli M et al. reported a higher prevalence of hard thyroid nodules in patients with acromegaly (56.7% vs. 33.7%), but it was not associated with an increase in malignancy rate, which could be explained by decreased elasticity due to fibrosis [9].

Long-lasting stimulation of the follicular epithelium by GH and IGF-1 can lead to an increase in thyroid volume and the development of goiter. GH and IGF-1 may also indirectly contribute to thyroid growth thereby increasing the effect of thyroid-stimulating hormone (TSH) [14]. Moreover, individuals with growth hormone deficiency have smaller thyroid volumes than normal subjects, which suggests that GH may play a permissive role in the growth of the thyroid gland [15]. Nodular thyroid disease is frequently seen in patients with acromegaly, especially in active disease [16]. Total thyroid volume increases [17] and increased thyroid size can be decreased by treatment with somatostatin analogues in acromegaly [18]. Thyroid follicular cells of patients with differentiated thyroid cancer (DTC) were considered to have a higher number of IGF-1 binding sites, and increased frequency of NRAS codon 61 point mutations was determined in DTC patients with acromegaly [19,20].

Thyroid cancer is one of the most common malignancies seen in patients with acromegaly [10], but there is still a debate about whether acromegaly is an independent risk factor for the development of thyroid cancer. According to a meta-analysis, the malignancy rate of the thyroid nodules in patients with acromegaly was not significantly higher than that of patients with nodular thyroid disease and without acromegaly [21]. Endocrine society guideline for acromegaly recommends thyroid ultrasonography in the presence of a palpable thyroid nodule [22]. The Acromegaly Consensus guidelines of the 11^th^ Acromegaly Consensus Conference also do not specifically recommend screening for thyroid cancer [1].

The reported sensitivity of different US-E techniques for the diagnosis of thyroid carcinoma ranges from 82%–100%, with specificity ranging from 81.1%–100% [23]. Although several studies indicate greater accuracy for thyroid cancer detection with US-E than conventional US, there is presently insufficient agreement among research groups regarding diagnostic criteria, and elastography is considered to be insensitive to diagnose some malignant tumor types [23–25].

US-E increases the accuracy of US in differentiating benign and malignant tumors during the evaluation of thyroid nodules, which can also be described as “electronic palpation”. ES and SI are seen to be bigger in malignant lesions [26]. Only one paper evaluated US-E in patients with acromegaly. Scacchi et al. evaluated the role of US-E to diagnose thyroid cancer in patients with acromegaly. The nodules in patients with acromegaly had higher elasto scores when compared to nodules in patients without acromegaly but with multinodular goiter (56.8% to 16%). Additionally, active acromegaly patients had a higher prevalence of hard nodules in comparison to the prevalence in cured or controlled patients. Although the hardness of thyroid nodules was higher, the malignancy rate was similar to the control group. The authors considered that increased stiffness was due to fibrosis as a result of increased synthesis of collagen and its apposition in the tissues by the excess of GH and IGF-1 [27–28]. We found similar results except for the similar prevalence of hard nodules in active and controlled acromegaly groups, however, we had a greater number of cases in our study.

Increased fibrosis possibly induced by GH and IGF-1 could be responsible for the stiffness in US-E, which may suggest that it is a benign process. Renal cortical stiffness determined by share wave elastography is related to parenchymal disease and fibrosis, which was explained by renal fibrosis [29]. Thyroid gland stiffness was increased in patients with acromegaly and associated with IGF-1 levels [17]. It is a known fact that histologically fibrotic nodules have higher stiffness on US-E evaluation [30]. According to our findings, decreased elasticity of nodules by US-E in acromegaly patients does not seem to estimate the malignancy of the nodules.

There may be some possible limitations to this study. First, the study was designed retrospectively. Second, the current study has a relatively small sample size. Finally, the BMI information of the patients in the control group is not available.

## 5. Conclusion

Thyroid nodules in acromegaly patients have higher elasto scores and the prevalence of hard nodules is higher in active disease. However, increased stiffness of nodules by US-E in acromegaly patients does not seem to estimate the malignancy of the nodules. Although elastography, as a noninvasive procedure, can assist traditional methods in the differential diagnosis of benign and malignant thyroid nodules [31], studies with more patients are needed to enlighten the importance of elastography in acromegaly-associated thyroid nodules.

## Figures and Tables

**Table 1:** Clinical characteristics and comorbidities of patients with acromegaly.

Patients, n	102
Female sex, n (%)	59 (58)
Current age (years)	55.09 ± 12.47
Age at diagnosis (years)	45.40 ± 13.48
Body mass index (kg/m^2^)	31.7 ± 6.1
Pituitary tumor size (mm)	17.13 ± 8.46
Pituitary tumor volume (mm^3^)	33.1 ± 31.22
Current IGF-1 (ng/mL)	212.07 ± 137.37
Current GH (mcg/L)	1.65 ± 2.60
Medical treatment (n = 89), n (%)	58/89 (65)
Patients received radiotherapy (n = 89), n (%)	16/89 (18)
Controlled disease^a^ (n = 90), n (%)	69/90 (76)
TSH (mIU/L)	1.48 ± 1.58
Free T4 **(**ng/dL)	1.07 ± 0.37
Nodule^b^	4.01 ± 2.87
Nodule size (mm)	9.40 ± 6.42
Elasto score	2.45 ± 0.63
Strain index	1.53 ± 0.73
Comorbidities (n = 102), n (%)	
Obesity (BMI > 30 kg/m2) (n = 62)	27 (43)
Diabetes mellitus	43 (42)
Hypertension	56 (54)
Hyperlipidemia	29 (28)
Coronary heart disease	14 (13)

a Controlled disease was defined as a fall in IGF-1 level to the normal range depending on age and sex.

b Mean number of thyroid nodules per patient

**IGF-1:** Insuline like growth factor -1; **GH:** growth hormone **TSH:** thyroid-stimulating hormone

**Table 2:** Ultrasound features of thyroid nodules in both groups.

	Acromegaly	Control group	p

**Nature**			
solid	155	100	0.928
mixed	11	5	0.608
cystic^a^	0	0	-

**Echogenicity**			
hypoechoic	16	13	0.552
isoechoic	26	18	0.867
hyperechoic	1	1	1
mixed	123	75	0.923

**Margins**			
regular	150	97	0.928
irregular	16	10	1

**Calcifications**			
absent			
micro	12	8	1
coarse	8	6	0.783

a Cystic nodules are not included because elastography could not be performed in these nodules.

**Table 3:** The prevalence of hard nodules in acromegaly patients and control group.

	ES 1–2	ES 3–4	P
**Acromegaly**	52%	48%	**<0.001**
**Control**	80%	20%	

ES: Elasto score

## Data Availability

The datasets used and/or analyzed during the current study are available from the corresponding author upon reasonable request.
